# Pain, distress, and anticipated recovery for older versus younger emergency department patients after motor vehicle collision

**DOI:** 10.1186/s12873-014-0025-y

**Published:** 2014-12-30

**Authors:** Gregory F Pereira, Samuel A McLean, Thomas J Tkacik, Robert A Swor, Jeffrey S Jones, David C Lee, David A Peak, Robert M Domeier, Niels K Rathlev, Phyllis L Hendry, Timothy F Platts-Mills

**Affiliations:** School of Medicine, University of Pennsylvania, Philadelphia, PA USA; Department of Anesthesiology, University of North Carolina, 101 Manning Drive, CB #7010, Chapel Hill, NC 27599-7010 USA; Departments of Emergency Medicine and Anesthesiology, University of North Carolina, 101 Manning Drive, CB #7010, Chapel Hill, NC 27599-7010 USA; Department of Emergency Medicine, William Beaumont Hospital, Royal Oak, MI USA; Department of Emergency Medicine, Spectrum Health – Butterworth Campus, Grand Rapids, MI USA; Department of Emergency Medicine, North Shore University Hospital, Manhasset, NY USA; Department of Emergency Medicine, Massachusetts General Hospital, Boston, MA USA; Department of Emergency Medicine, St. Joseph Mercy Hospital, Ann Arbor, MI USA; Department of Emergency Medicine, Baystate Medical Center, Springfield, MA USA; Department of Emergency Medicine and Pediatrics, University of Florida-Jacksonville, Jacksonville, FL USA

**Keywords:** Geriatrics, Pain, Emergency medicine, Traffic accidents

## Abstract

**Background:**

Motor vehicle collisions (MVCs) are the second most common injury mechanism resulting in emergency department (ED) visits by older adults. MVCs result in substantial pain and psychological distress among younger individuals, but little is known about the occurrence of these symptoms in older individuals. We describe the frequency of and characteristics associated with pain, distress, and anticipated time for physical and emotional recovery for older adults presenting to the ED after MVC in comparison to younger adults.

**Methods:**

In-person interviews were conducted for adults presenting to one of eight EDs after MVC without an obvious fracture or injury requiring admission as part of two prospective studies. Pain severity was assessed using a 0–10 verbal scale. Distress was assessed using the Peritraumatic Distress Inventory (range 0–52). Patients were asked to estimate their expected time for physical and emotional recovery; these responses were dichotomized to <30 or ≥30 days. ED pain and distress and associations between patient and collision characteristics and ED pain and distress were examined for patients age 65 years and older and patients age 18 to 64.

**Results:**

Older (n = 96) and younger (n = 943) adults had the same mean pain scores (5.5, SD 2.5 vs. 5.5, SD 2.4). Distress scores were lower in older than in younger adults (15.5, SD 9 vs. 19.2, SD 10). A higher percentage of older adults than younger adults had an anticipated time to physical recovery ≥30 days (41%, 95% confidence interval [CI] 28%-55% vs. 11%, 95% CI 9%-13%). Similarly, older adults were more likely to have an anticipated time for emotional recovery ≥30 days (45%, 95% CI 35%-55% vs. 17%, 95% CI 15%-20%). Older adults were less likely than younger adults to have moderate or severe neck pain (score ≥4) (25%, 95% CI 23% to 41% vs. 54%, 95% CI 48% to 60%) or back pain (31%, 95% CI 23% to 46% vs. 56%, 95% CI 51 to 62%) but more likely to have moderate or severe chest pain (42%, 95% CI 32% to 50% vs. 20%, 95% CI 16 to 23%). Pre-MVC depressive symptoms and pain catastrophizing were positively associated with pain and distress in both older and younger adults.

**Conclusions:**

In our cohort, older adults who presented to the ED after MVC experienced similar pain severity as younger patients and less distress but were more likely to estimate their times for physical and emotional recovery to be 30 days or more. Increased emergency provider awareness of acute pain and distress symptoms among older patients experiencing MVC may improve outcomes for these patients.

## Background

Adults age 65 years and older make an estimated 250,000 US emergency department (ED) visits each year for evaluation after motor vehicle collision (MVC), making this the second most common cause of injury resulting in ED visits for this age group [1,2]. The number of older adults experiencing an MVC is anticipated to double between 2010 and 2030 [3]. Prolonged hospital stays and high mortality rates have been described for older adults experiencing injuries requiring admission after MVC [4-6], and high rates of acute pain and distress have been described for individuals of all ages with severe injuries [7,8]. However, 80% of older adults who present to the ED after MVC are discharged to home after evaluation [9], and outcomes for these patients have received little study.

Among younger adult MVC patients who are discharged to home after ED evaluation, acute pain and distress symptoms are common [10]. These symptoms cause substantial suffering, and are also important predictors of persistent pain and psychological sequelae after MVC, which constitute an important post-injury public health problem [11]. While the epidemiology of acute pain and psychological symptoms has been described in younger adults, initial pain and psychological symptoms in older adults presenting to the ED after MVC have not been well characterized. Understanding age-related differences in acute pain and psychological symptoms following MVC has the potential to help providers anticipate the types and severity of problems older patients experience after MVC. In addition, examining associations between patient age, acute pain, and psychological symptoms may provide insights into the mechanisms underlying the initial response to injury across the lifespan and the influence of this response on the development of persistent post-MVC, which is a major public health problem in developed countries [11]. Early analgesic treatment and education regarding movement and pain-relief can improve outcomes after MVC [12,13], but further work is needed to identify high risk patients and to understand mechanisms leading to persistent pain and psychological sequelae.

The objective of this study is to compare pain, distress symptoms, and recovery expectations between older and younger adults who present to the ED after MVC and are discharged to home, and to examine associations between patient and collision characteristics and pain and distress symptoms for these two age groups.

## Methods

### Study design and setting

We analyzed cross-sectional data obtained as part of two prospective cohort studies of patients evaluated in the ED following MVC. The two studies, European American CRASH (EA CRASH) and Older Adult CRASH (OA CRASH), each enrolled patients from the same eight EDs in four no-fault insurance states (Michigan, Massachusetts, New York and Florida), where litigation associated with MVC is relatively uncommon. Details of the methods for EA CRASH have been reported [14]. A summary of the methods and differences between the two cohorts is presented below. Each study was approved by the Institutional Review Boards of the coordinating institution (University of North Carolina) and all participating hospitals (William Beaumont Health System, Spectrum Health System. North Shore University Hospital, Massachusetts General Hospital, St. Joseph Mercy Hospital, Baystate Medical Center, University of Florida Health System), and each participant provided written informed consent.

### Selection of participants

**Figure 1:**
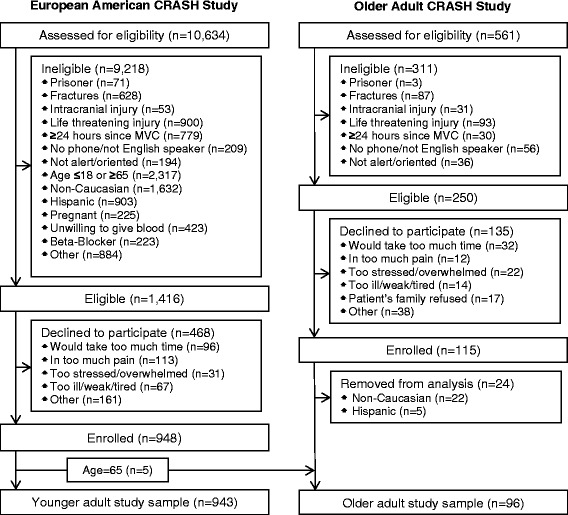
**Flow diagrams of eligibility assessment and enrollment.** Reasons for ineligibility and the patient’s race and ethnicity in the Older Adult CRASH Study are not mutually exclusive. Five of the patients in European American CRASH were age 65. These patients were analyzed in the age 65 and older group.

Patients who presented to the ED within 24 hours of an MVC with injuries unlikely to require admission were screened for eligibility. EA CRASH enrollment was limited to non-Hispanic European American adults age 18 to 65 years. Exclusions based on self-reported race and ethnicity were made in EA CRASH in order to prevent population stratification bias associated with genetic analyses performed as part of the study [15]. OA CRASH included individuals of all races and ethnicities age 65 years and older. In both studies, patients were excluded if they were unable to read and understand English, were not alert and oriented, had fractures that were evident at the time of the ED assessment by the research assistant, intracranial injury, laceration with significant hemorrhage, or other injuries considered life-threatening or likely to require hospital admission as judged by the treating physician at the time of RA assessment. In OA CRASH, patients were also excluded if they had cognitive impairment as defined by a Six-Item Screener score less than four [16]. In the study of younger adults (EA CRASH), patients taking a β-receptor antagonist or taking daily opioids were also excluded. During the five months for which recruitment to EA CRASH and OA CRASH overlapped at the eight study sites, patients age 65 years were first assessed for enrollment in EA CRASH. In order to define comparable older and younger groups of participants, we excluded from analysis OA CRASH patients who were non-white or Hispanic. Patients 65 years of age who were enrolled in the EA CRASH study (n = 5) were categorized as age 65 years and older and analyzed with OA CRASH patients (Figure 1).

**Figure 2:**
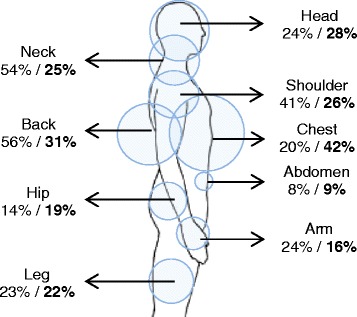
**Frequency of moderate or severe pain (pain score ≥4) by region for adults age 18–64 years vs. adults age ≥65 years.** Percentages for older patients are in bold. Areas of circles are proportional to frequency of pain among older individuals.

### Measures

Study participants were interviewed by trained research assistants in the ED using a standardized questionnaire with explicit definitions of study variables. These interviews assessed sociodemographic and crash characteristics, pain, and psychological symptoms (e.g., distress) and traits (e.g., pain catastrophizing, depressive symptoms). The severity of motor vehicle damage was rated by study participants as minor, moderate, or severe, with severe damage defined as the vehicle not being drivable. Agreement between ED patient descriptions of the severity of vehicle damage and police reports have previously been described [17]. Pain severity in the ED and average pain in the past month were assessed using a 0 to 10 verbal scale. Distress was assessed using the Peritraumatic Distress Inventory, a 13-item measure intended to assess distress in the early aftermath of trauma [18]. Anticipated time to physical recovery and anticipated time to emotional recovery were assessed in days. Pain catastrophizing, defined as cognitive and emotional responses to pain characterized by magnification of pain, rumination on pain, and feelings of helplessness in response to pain, was assessed using the 13-item Pain Catastrophizing Scale [19]. Pain catastrophizing was dichotomized based on a Pain Catastrophizing Score of ≥10 (range 0–52); this score represented the 75^th^ percentile of scores for the entire cohort.

In the study of younger adults, depressive symptoms were measured using the 20-item Center for Epidemiologic Studies Depression Scale-Revised (CES-D-20) [20]. In the study of older adults, depressive symptoms was measured using a 10-item version of the Center for Epidemiologic Studies Depression Scale (CES-D-10) adapted specifically for older adults (n = 41) [21]) or a two item measure of depression which assesses dysthymia and anhedonia symptoms (n = 50) [22]. Agreements between each of the three instruments used to assess for the presence of depressive symptoms and a criterion standard diagnosis of depression established using the National Institute of Mental Health Diagnostic Interview Schedule have been described [23]. The presence of depressive symptoms was determined based on previously reported cutoffs for each of the three measures used: ≥16 for the CES-D-20 measure (range 0–60) [20]; ≥4 for the CES-D-10 score (range 0–10) [21]; or a yes to either question from the two-item instrument [22]. Use of diagnostic imaging tests, diagnosis of fractures, and disposition from the ED (i.e. discharged, observed, or admitted) were ascertained by research assistants from each study site during the week following the ED visit. Details of the data extraction process have been published [14] and were the same for both studies.

### Data analysis

Sociodemographic characteristics of participants age 65 years and older (older adults) and those age 18 to 64 years (younger adults) were summarized using proportions for categorical variables and means and standard deviations for continuous variables. Anticipated times to physical and emotional recovery were non-normally distributed. Most patients anticipated that they would recover in the first 14 days; a second cluster of patients anticipated physical and emotional recovery ≥30 days. Based on this distribution, anticipated times to physical and emotional recovery were dichotomized into less than 30 days vs. 30 days or more. Pain scores and distress symptom scores among subgroups of patients defined by sociodemographic, psychological, and collision characteristics are reported as means and associated 95% confidence intervals separately for older and younger adults. The extent to which associations between patient characteristics and outcomes differed between younger and older adults was tested using linear regression models including variables for 1) age group, 2) the patient or collision characteristic, and 3) a term for the interaction between the age group and the patient or collision characteristic. A p-value <0.05 for the interaction term was defined as indicating a statistically significant interaction, indicating that the relationship between the characteristic and the outcome was different for younger vs. older adults. All available data were used for analyses, and no sample size calculation was performed. Analyses were conducted using Stata IC 11.0 (StataCorp LP, College Station, Texas).

## Results

The EA CRASH study screened 10,634 adults between February 2009 and October 2011; OA CRASH screened 561 adults between June 2011 and March 2013. From these two studies, 948 adults from EA CRASH and 91 adults from OA CRASH met eligibility criteria, consented to participation, and met subsequent criteria for these analyses (Figure 1). After reclassifying 5 patients age 65 years from EA CRASH as older adults, analyses were then conducted on 943 individuals age 18 to 64 years and 96 individuals age 65 years or older.

Relative to younger adults, older adults had less formal education, worse self-rated health, and were more likely to report pain during the month prior to the MVC (Table 1). The majority of older and younger patients were drivers, wore seat belts, and reported moderate or severe vehicle damage. Older adults were more likely to report airbag deployment, likely because they were more often involved in head-on or side-impact collisions. Older adults were also more likely to be transported by ambulance, and to report a greater sense of life threat during the collision. Computed tomography was used more often than plain radiography to image the cervical spine in older adults (Table 2). Overall, plain radiographs and computed tomography scans were both performed more frequently for older adults than younger adults. Clinically apparent fractures were exclusion criteria for both studies; the diagnosis of fractures after enrollment was more common among older than younger adults, but occurred in less than ten percent of adults in both groups. Older adults were more likely than younger adults to be admitted (13% vs. 1%) or observed (8% vs. 1%) than younger adults.

**Table 1:** **Characteristics of patients presenting to the emergency department after motor vehicle collision, by age group (years)**

**Characteristic**	**Age 18–64**	**Age ≥65**
	**N = 943**	**N = 96**
Age, mean (SD), years	35 (13)	72 (6)
Female, %	60	52
Education, %		
8-11 years	4	12
High school	19	21
Post high school*	39	29
College graduate	25	20
Post graduate	12	18
General health, %		
Excellent	31	18
Very good	41	34
Good	22	29
Fair	7	16
Poor	1	3
Average pain past month, %		
None (0)	65	44
Mild (1–3)	15	24
Moderate (4–6)	12	17
Severe (6+)	9	16
≥4 drinks per week, %	39	17
Pain catastrophizing, %	44	33
Depressive symptoms, %	20	25
Driver, %	86	84
Seat-belt, %	90	93
Collision type^†^, %		
Head-on	57	48
Side-impact	34	44
Rear-ended	36	27
Air bags deployed, %	29	53
Damage severity, %		
Minor	14	13
Moderate	31	48
Severe	55	39
Life threat^ǂ^ (0–10), mean (SD)	4.2 (3.1)	4.9 (3.6)
Arrived by ambulance, %	58	88

*Includes technical schools and college without graduation.

^†^Not mutually exclusive.

^ǂ^How life threatening was your motor vehicle accident?

**Table 2:** **Radiographic imaging use, fractures, and disposition, by age group**

**Event**	**Age 18–64**	**Age ≥65**
	**N = 943**	**N = 96**
Plain radiography		
Cervical spine, %	27	6
Chest, %	32	43
Pelvis, %	9	14
Total radiographs, mean(SD)	1.2 (1.1)	1.6 (1.1)
Computed tomography (CT) scans		
Head, %	24	45
Cervical spine, %	23	36
Chest, %	5	19
Abdomen/pelvis, %	8	17
Total CT scans, mean(SD)	0.7 (1.0)	1.4 (1.4)
Fractures, %		
Spine	0	2*
Rib	<1	3
Sternum	<1	2
Other	<1	1
Disposition, %		
Discharged	98	79
Observation	1	8
Admitted	1	13

*Includes one patient with a compression deformity of the sixth thoracic vertebrae the age of which was unknown.

Mean pain scores in older and younger adults were identical (Table 3). Moderate or severe pain in one or more body region was reported by 77% (95% confidence interval [CI], 67% to 84%) of older adults and 80% (95% CI, 77% to 82%) of younger adults. Both older and younger patients reported a median of 3 (IQR 2–5) body regions with pain (pain score ≥1). The distribution of body regions with pain differed between older and younger adults. Moderate or severe chest pain was reported by 42% (95% CI, 20% to 38%) of older adults compared to 20% (95% CI, 17% to 22%) of younger adults (Figure 1). In contrast, neck and back pain were reported by 25% (95% CI, 17% to 34%) and 31% (95% CI, 23% to 41%) of older adults, respectively, compared to 54% (95% CI, 50% to 57%) and 56% (95% CI, 53% to 60%) of younger adults, respectively.

**Table 3:** **Pain, distress, and anticipated recovery after motor vehicle collision, by age group**

	**Age 18–64**			**Age ≥65**		
**Characteristic**	**N = 943**			**N = 96**		
Pain severity, mean (95% CI)		5.5	(5.3–5.7)		5.5	(5.0–6.0)
Distress, mean (95% CI)*		19	(19–20)		16	(14–17)
Anticipated time for physical recovery ≥30 days, % (95% CI)^†^		11	(9–13)		41	(28–55)
Anticipated time for emotional recovery ≥ 30 days, % (95% CI)		17	(15–20)		45	(35–55)

*Distress measured using the Peritraumatic Distress Inventory scale.

^†^N = 51 for patients age ≥65. Mark on X-axis of each histogram indicates 30 days.

Mean distress scores were slightly lower in older adults than in younger adults (15.5, 95% CI 14 to 17 vs. 19.2, 95% CI 19 to 20). However, the prevalence of substantial distress (distress score ≥ 13 [24]) was nevertheless high in both groups, with half of older adults and 68% of younger adults experiencing substantial distress. A higher percentage of older adults than younger adults reported an anticipated time to physical recovery of 30 days or more (41%, 95% CI 28%-55% vs. 11%, 95% CI 9%-13%). Similarly, a higher percentage of older adults reported an anticipated time for emotional recovery of 30 days or more (45%, 95% CI 35%-55% vs. 17%, 95% CI 15%-20%).

Among younger adults, females and those with less formal education had higher rates of pain and distress (Tables 4, 5). Trends in these relationships were also observed among older adults. For both younger and older adults, patients with higher reported pre-MVC depressive symptoms and higher pain catastrophizing in the ED had higher mean pain and distress scores than those that did not. Younger adults who were not rear-ended had higher distress scores than those who were rear-ended; this association was not observed in older adults. This interaction between age category and rear-end collision on the outcome of distress was statistically significant (p < 0.01); no other interactions between age category and the characteristics examined in Tables 4 and 5 were statistically significant or were suggested by visual inspection of the results.

**Table 4:** **Mean emergency department pain scores (0–10 scale) for younger and older adults by patient and collision characteristics**

	**Age 18-64**		**Age ≥65**	
**Characteristic**	**N**	**Mean (95% CI)**	**N**	**Mean (95% CI)**
Sex				
Female	565	5.7 (5.5,5.9)	52	5.6 (4.9,6.3)
Male	369	5.3 (5.0,5.5)	44	5.4 (4.6,6.3)
Education				
8-11 years	42	7.1 (6.3,7.9)	11	6.2 (4.6,7.8)
High school	180	5.8 (5.5,6.1)	20	5.4 (4.3,6.4)
Post high school	365	5.9 (5.7,6.1)	28	6.2 (5.1,7.2)
College grad	234	5.0 (4.7,5.3)	29	5.5 (4.5,6.4)
Post grad	111	4.4 (4.0,4.8)	17	4.3 (3.2,5.4)
Pain catastrophizing				
Yes	409	6.1 (5.9,6.3)	31	6.4 (5.5,7.2)
No	513	5.1 (4.9,5.3)	63	5.1 (4.4,5.7)
Depressive symptoms				
Yes	191	6.0 (5.6,6.3)	22	6.5 (5.6,7.5)
No	741	5.4 (5.2,5.6)	68	5.0 (4.4,5.6)
Driver				
Yes	801	5.5 (5.3-5.6)	79	5.4 (4.8,5.9)
No	133	5.9 (5.5-6.3)	15	6.1 (4.5,7.8)
Rear-ended				
Yes	334	5.5 (5.2,5.8)	26	5.6 (4.6,6.5)
No	600	5.6 (5.4,5.7)	68	5.5 (4.8,6.1)
Damage severity				
Severe	500	5.5 (5.3,5.7)	35	5.3 (4.5,6.1)
Moderate	276	5.6 (5.3,5.9)	43	5.7 (5.0,6.5)
No or minor	127	5.2 (4.7,5.6)	11	4.2 (2.6,5.8)

**Table 5:** **Mean emergency department distress scores (0–52 scale) for younger and older adults by patient and collision characteristics**

	**Age 18-64**		**Age ≥65**	
**Characteristic**	**N**	**Mean (95% CI)**	**N**	**Mean (95% CI)**
Sex				
Female	560	21.1 (20.3,21.9)	52	16.9 (14.5,19.3)
Male	369	16.3 (15.3,17.2)	44	13.9 (11.2,16.7)
Education				
8-11 years	42	24.4 (21.3,27.5)	11	17.4 (11.9,22.9)
High school	178	20.2 (18.8,21.7)	20	14.0 (9.65,18.4)
Post high school	360	19.9 (18.9,20.9)	28	15.8 (12.4,19.2)
College grad	235	17.6 (16.3,18.9)	29	17.5 (13.6,21.5)
Post grad	112	16.9 (15.3,18.5)	17	13.0 (9.6,16.5)
Pain catastrophizing				
Yes	408	22.0 (21.2,23.0)	32	19.9 (17.0,22.7)
No	510	16.8 (16.0,17.7)	64	13.4 (11.2,15.5)
Depressive symptoms				
Yes	190	22.7 (21.2,24.2)	23	18.9 (15.8,21.9)
No	737	18.3 (17.6,19.0)	69	14.1 (11.9,16.2)
Driver				
Yes	801	19.4 (18.7,20.1)	79	15.3 (13.3,17.3)
No	133	17.7 (16.0,19.4)	15	16.8 (12.3,21.3)
Rear-ended				
Yes	337	17.0 (16.0,18.0)	26	17.5 (13.5,21.5)
No	592	20.4 (19.6,21.2)	70	14.8 (12.8,16.8)
Damage severity				
Severe	497	21.2 (20.3,22.1)	35	16.5 (13.2,19.8)
Moderate	277	18.0 (16.8,19.1)	43	15.2 (12.6,17.8)
No or minor	125	14.1 (12.5,15.7)	11	13.9 (9.0,18.8)

Sensitivity analyses were conducted in which the sample of older adults was further restricted in order to make them more similar to the younger cohort. Among the subset of older adults who were not taking daily opioids prior to the collision, did not have a fracture, and were discharged home (n = 68), the mean pain score (5.1, 95% CI 4.5 to 5.7) and mean distress score (12.9, 95% CI 10.6 to 15.2) were similar to scores for the overall sample of older adults. Comparisons between these scores and scores for younger patients did not change the overall findings that older patients had similar pain scores, lower distress scores, and were more likely to have an anticipated time for physical recovery or emotional recovery of 30 days or more when compared to younger adults.

### Limitations

We compare results from two studies with minor differences in inclusion and exclusion criteria and assessment measures. In the study of younger adults, 5% of patients were excluded because they were unwilling to provide a blood sample; this was not an exclusion criterion for the older sample. The OA CRASH study used different instruments for assessing depressive symptoms than EA CRASH. Although the accuracies of each of these three instruments for identifying depressive symptoms when compared to a criterion standard diagnosis of depression is good or excellent (area under ROC >0.8 for each of the three measures vs. criteria standard [23]), it is possible that the use of different measures caused different estimates for the frequency of depression or associations between depression and pain and distress for older vs. younger adults.

Only 67% and 46% of eligible patients participated in the EA CRASH and OA CRASH studies, respectively. Among eligible patients, reasons for non-participation were similar for younger and older adults. For both studies, some patients declined to participate because they were either in too much pain, were too overwhelmed or stressed, or were too weak, ill, or tired. The total number of patients who decline participation for any of these reasons was 15% of eligible patients in the EA CRASH study and 19% of eligible patients in the OA CRASH. Non-enrollment of these and other patients likely creates some selection bias, but whether selection bias due to eligible patients declining to participate results in over- or under-estimates of pain and distress symptoms is unknown. Also, only non-Hispanic Caucasian patients were enrolled in EA CRASH, and the analysis of participants in OA CRASH was restricted to non-Hispanic Caucasians. The experiences of pain and distress and the effect of age on these experiences may be different in other racial and ethnic groups [25].

## Discussion

Adults age 65 and older are a growing injury population [26-28], but the types of problems faced by older adults after common injury mechanisms have not been well characterized. In this prospective study of adults presenting to the ED with minor injuries due to MVC, we observed that acute pain was as much a problem for older adults as for younger adults, with more than 75% of patients in both age groups experiencing moderate or severe pain. In addition, while average distress scores were lower in older adults than younger adults, more than half of both older and younger adults presenting to the ED after MVC experienced substantial distress symptoms.

Persistent pain after MVC is a major public health problem and acute pain is the strongest single risk factor for persistent pain [29]. Our findings of similar acute pain scores among older and younger adults suggests that persistent pain is likely to be at least as common among older adults as among younger adults. Our finding of a mean pain score of 5.5 in the older adults is also consistent with nationally-representative data, in which 61% of older adults who were discharged after an MVC-related ED visit had moderate or severe pain [1]. In addition, the majority of both younger and older patients had moderate or severe pain for each damage severity category, a finding that is consistent with prior research indicating that acute pain severity is largely independent of the severity of the collision [29, 30]. Further studies which evaluate chronic pain outcomes and predictors of chronic pain among older adults are needed, as are studies which evaluate etiologic mechanisms of chronic pain in both groups.

Posttraumatic stress disorder (PTSD) is another common and morbid health problem resulting from MVC [31], and the initial psychological response to MVC is an important indicator of PTSD risk following MVC [32-34]. PTSD is also known to be prevalent in approximately 1% of older adults [35], and advanced age may exacerbate symptoms of PTSD [36]. In addition, the acute stress response that results from the experience of life-threat may be an important mechanism contributing to persistent post-MVC pain [37]. Although the problem of PTSD after MVC has not been described among older adults experiencing MVC, other injuries, such as falls, are known to cause PTSD in older adults [38,39]. Our results suggest that substantial distress is experienced by more than half of both older and younger adults presenting to the ED after MVC and suggest that PTSD is a problem in older adults. Interventions to treat the acute psychological response to MVC (e.g. cognitive-behavioral interventions) might be efficacious in reducing both persistent pain-related disability [40] and psychological sequelae [41,42] in both age groups.

Older adults were more likely than younger adults to have an anticipated time to physical recovery or emotional recovery of 30 days or more. Patient’s expectations for health outcomes are correlated with and likely influence actual health outcomes [43], and evidence from observational studies of other types of musculoskeletal pain indicates that older adults typically do require more time to recover than younger patients [44,45]. Thus, it seems likely that the differences in anticipated recovery times between older and younger patients in our study result largely from accurate patient assessments of the actual time that they will need to recover.

In our sample, pain catastrophizing was strongly associated with both pain severity and distress symptoms among both younger and older adults. Pain catastrophizing has previously been associated with pain severity among adults with spinal cord injury [46] and associated with pain severity and function among older adults with osteoarthritis [47]. Further, decreases in pain catastrophizing during the course of a multi-component intervention to treat chronic pain were associated with decreases in pain severity and disability [48]. Depression was also associated with pain severity and peritraumatic distress among older and younger adults in our study. Whether interventions to reduce pain catastrophizing or depression can improve pain and functional outcomes for patients presenting to the ED after MVC is unknown.

Among younger adults, those with less formal education had more pain and distress; this relationship was previously described for a subset of this cohort [49]. Increased pain and distress among less educated patients may be because these patients have less understanding of the nature of injury, less self-efficacy or more limited coping skills, or an increased burden of financial stress from MVC. The data for older adults suggest a similar inverse relationship between educational attainment and pain and distress. Further studies to better understand factors accounting for increased acute pain and distress among ED patients with lower socioeconomic status are needed.

A greater proportion of older adults than younger adults experienced moderate or severe chest pain and fewer experienced moderate or severe back or neck pain. Persistent neck, shoulder, and back pain after MVC (i.e. whiplash syndrome) is a well described phenomenon [50]. It is unclear whether acute chest pain after MVC leads to a persistent pain condition in older adults. Five of the 40 older adults in the study with moderate or severe chest pain were found to have rib or sternal fractures. The cause of chest pain in the remaining 35 patients is not known, but some likely had radiographically-occult rib fractures.

More older adults came to the ED via ambulance than younger adults. Contrary to our expectations, recent work by our group does not support the presence of a lower threshold for ambulance transport for older adults experiencing MVC [51]. Ambulance transport can be a stressful experience for patients [52], but also provides an opportunity for prehospital treatment. Prehospital care may have affected pain and distress symptoms in the study sample, but existing evidence suggests that older adults are less likely to receive analgesics than younger adults during prehospital care [53]. Further studies are needed which examine the influence of prehospital care on longitudinal pain outcomes among older adults experiencing MVC.

Prior studies have characterized outcomes after minor blunt trauma in older adults, but have included a large proportion of patients who presented to the ED after a fall [54,55]. MVCs are distinct from falls because the older adults who experience MVC are, on average, higher functioning and more likely to be living independently than patients who fall [56]. Further, our results indicate that acute pain is a substantial problem among older adults who present to the ED after MVC; acute pain is less common after a fall and when present usually results from a long bone fracture. We observe that acute pain is common among older adults receiving emergency care after MVC. Further understanding of long-term outcomes after MVC among older adults and the factors which improve and impede the recovery process after MVC are needed to guide the initial care of this growing and vulnerable trauma population. Understanding the long-term impact of MVC on older adults also has the potential to inform the ongoing debate regarding driver safety among older adults [57,58].
